# Obesity and increased burden of hip and knee joint disease in Australia: Results from a national survey

**DOI:** 10.1186/1471-2474-13-254

**Published:** 2012-12-20

**Authors:** Ilana N Ackerman, Richard H Osborne

**Affiliations:** 1 Melbourne EpiCentre, Department of Medicine (Royal Melbourne Hospital), The University of Melbourne, Melbourne, Australia; 2 Public Health Innovation, Population Health Strategic Research Centre, Deakin University, Melbourne, Australia

**Keywords:** Obesity, Osteoarthritis, Quality of Life, Health status

## Abstract

**Background:**

Research involving more representative samples is needed to extend our understanding of the broader impact of obesity in hip or knee joint disease (arthritis and OA) beyond clinical settings. Although population-based research has been conducted in the United States, how these findings translate to other countries is unclear. Using a national approach, this study explored associations between obesity and the burden of hip and knee joint disease in Australia (in terms of prevalence, pain, stiffness, function, Health-Related Quality of Life (HRQoL) and disease severity).

**Methods:**

A random sample of 5000 Australians (≥39 years) from the federal electoral roll was invited to complete a mailed questionnaire to identify doctor-diagnosed hip arthritis, hip OA, knee arthritis and knee OA and evaluate the burden of these conditions. Validated questionnaires included the WOMAC Index, Assessment of Quality of Life instrument and Multi-Attribute Prioritisation Tool. Body Mass Index (BMI) was classified into underweight/normal weight (≤24.99 kg/m^2^), overweight (25–29.99) or obese (≥30). Multiple logistic regression was used to estimate odds of arthritis and OA, with demographic and socioeconomic variables included in the models. Associations between BMI and other variables were investigated using analysis of covariance, with adjustment for age and sex.

**Results:**

Data were available from 1,157 participants (23%). Overweight participants had increased odds of knee arthritis (adjusted OR (AOR) 1.87, 95%CI 1.14-3.07) and knee OA (AOR 2.11, 95%CI 1.07-4.15). Obesity was associated with higher prevalence of hip arthritis (AOR 2.18, 95%CI 1.17-4.06), knee arthritis (AOR 5.47, 95%CI 3.35-8.95) and knee OA (AOR 7.35, 95%CI 3.85-14.02). Of those with arthritis or OA, obese individuals reported more pain (for hip arthritis, hip OA and knee OA), greater stiffness (for hip arthritis, knee arthritis and knee OA), worse function (all diagnoses), lower HRQoL (for hip arthritis and hip OA) and greater disease severity (all diagnoses).

**Conclusions:**

This national study has demonstrated that the odds of arthritis and OA was up to 7 times higher for obese individuals, compared with those classified as underweight/normal weight. Concurrent obesity and joint disease had a marked impact on several key aspects of wellbeing, highlighting the need for public health interventions.

## Background

Hip and knee joint disease (arthritis and osteoarthritis (OA)) are ongoing public health challenges internationally, which are set to worsen with ageing populations. The number of hip and knee replacements in Australia has almost doubled in the past decade and over 80,000 procedures are now undertaken annually, predominantly for OA [1]. In recent years, the prevalence of obesity has also risen substantially in many countries including Australia, Canada and the United States (US) [2], with 61% of Australian adults considered to be overweight or obese [3]. Overweight and obesity represent the third highest risk factors for the burden of illness in Australia, after smoking and hypertension [3].

Obesity is thought to contribute to the development and progression of OA through increased mechanical load and more recently, via hormonal and metabolic mechanisms [4,5]. There is some evidence to support a relationship between obesity and risk of hip OA [6], and several large studies have shown a clear link between obesity and risk of knee OA [7-12]. To date, the association between obesity and burden of hip and knee joint disease (in terms of prevalence and impact) has not been explored in Australia. Specific data pertaining to the hip and knee joints are not available from Australian population-based studies, including the triennial National Health Survey and state-based health surveys, which have collected data on self-reported arthritis and OA affecting any joint/s [13-16]. Nonetheless, two studies from single Australian states have identified a relationship between increasing Body Mass Index (BMI) and risk of non-specific arthritis. Analysis of data from the Victorian Population Health Survey showed that overweight individuals were 60% more likely to have arthritis and those who were obese had twice the risk [13]. A similar relationship between obesity and risk of general arthritis was also evident from the South Australian Health Omnibus Survey [14].

Information on obesity as both a risk factor and a determinant of quality of life is important for understanding how to deal with hip and knee joint disease at a population level. However, many studies investigating the relationship between obesity and the impact of hip and knee OA have been conducted in clinical settings, with limited generalisability. Overweight and obesity have been associated with impaired quality of life in a primary care study of patients with hip or knee OA [17] and most recently, computer simulation models demonstrated a substantial expected loss of quality-adjusted life years for obese individuals with knee OA [18]. Australian research involving obese adults recruited from gastric banding and weight loss programs found that a knee OA subgroup had poorer physical functioning, increased bodily pain and lower Health-Related Quality of Life (HRQoL), compared to those without OA [19]. Clinical studies and those involving convenience samples have also found obesity to be associated with increased pain [20-22], worse health status [22] and greater disability in hip or knee OA [22-24], although the applicability of these findings to other settings is not clear.

Research using more representative samples is needed to extend our understanding of the personal impact of concurrent obesity and hip or knee joint disease beyond primary care and outpatient settings. The use of population-based sampling enables recruitment of people with a range of disease severity, including those with less severe joint disease who may not be receiving treatment and are therefore unlikely to be included in clinical studies. This is important for ensuring that research findings are relevant across the disease severity spectrum, and not limited to only those with more severe joint disease. Additionally, population-based sampling facilitates the recruitment of people from varied socioeconomic backgrounds, representing an advance over clinical studies which commonly comprise patients from a limited catchment area. To date, our understanding of the relationship between obesity and personal impact of arthritis or OA comes predominantly from population-based research conducted in the US [25-29]. Most studies in this area have focused on functional status, disability and activity limitations, although other population-based studies (for example, those investigating racial differences in pain among people with knee OA [30,31]) have included Body Mass Index (BMI) as part of a range of covariates. Whether the impact of obesity is similar in other countries is not known and further investigation of the relationships between obesity and pain, function and HRQoL in hip and knee joint disease in other populations is warranted.

Using a national approach, this study aimed to:

1. investigate associations between obesity and the prevalence of hip and knee joint disease (arthritis and OA) in Australia; and

2. explore associations between obesity and pain, stiffness, physical function, HRQoL and disease severity among people with hip or knee joint disease.

## Methods

### Study design

This paper reports the results of a national cross-sectional survey.

### Participants

As electoral enrolment and voting is compulsory for Australians aged 18 years and over, the federal electoral roll provides comprehensive coverage of the Australian adult population. Medical researchers can apply for an electoral roll extract for use in research or public health screening programs. In May 2009, following approval by The University of Melbourne Human Research Ethics Committee and the Australian Electoral Commission, we obtained an extract which included the names, age group, sex and postal address details for a random sample of 10,000 individuals drawn from all federal electoral divisions. This extract was used to randomly select a sample of the Australian population aged 39 years and over from all states and territories (N=5,000), stratified by age range category. The lowest two age categories (39–43 years and 44–48 years) were over-sampled to increase precision, as the prevalence of arthritis is lower among younger people [13,32].

### Procedure

**Figure 1:**
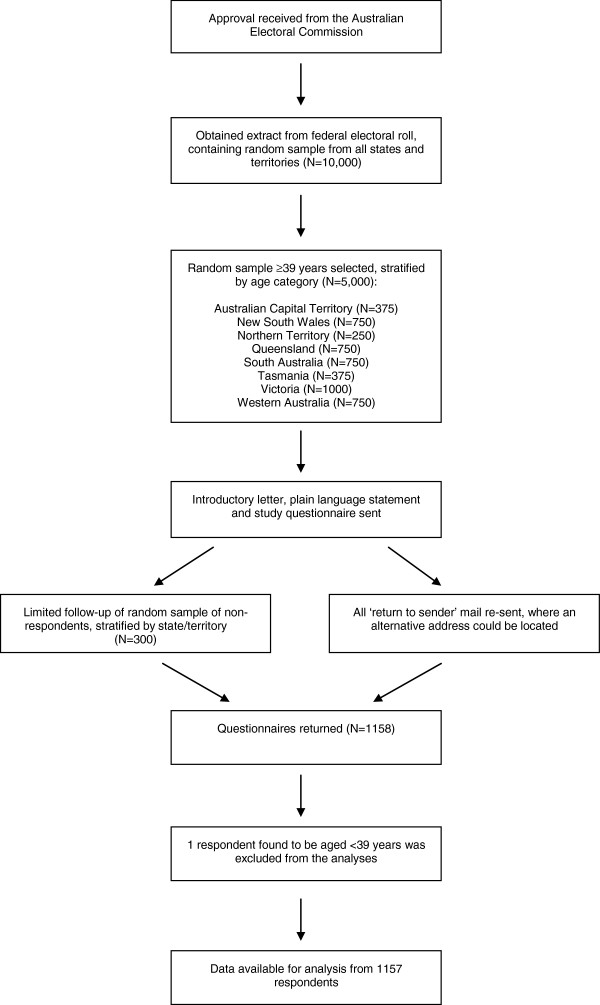
Sample selection and recruitment.

In June 2009, an introductory letter, plain language statement and questionnaire were mailed to the selected sample (Figure 1). Reply-paid envelopes were provided to maximize response rates. To minimise participant burden and maximise response rates, return of a completed questionnaire was deemed to constitute consent. This procedure was approved by The University of Melbourne Human Research Ethics Committee. Limited follow-up with repeat administration of the study questionnaire was undertaken for a random sample of non-participants approximately 6 weeks after the initial mailing (stratified by state/territory, *n*=300). All questionnaires marked ‘return to sender’ were re-sent where an alternative postal address could be located.

Although hip OA and knee OA were the primary conditions of interest, previous studies have indicated that individual knowledge of arthritis type can be limited [16,33]. The questionnaire was therefore designed to screen for the presence of doctor-diagnosed hip OA and knee OA *and* doctor-diagnosed hip arthritis and knee arthritis to capture the majority of possible OA cases. Screening was performed using self-reported measures, similar to the methodology used by the US Centers for Disease Control [33]. Participants were asked:

“have you ever been told by a doctor or other health professional that you have hip arthritis?”;

“have you ever been told by a doctor or other health professional that you have hip osteoarthritis?”;

“have you ever been told by a doctor or other health professional that you have knee arthritis?”; and

“have you ever been told by a doctor or other health professional that you have knee osteoarthritis?”

Individuals who reported having x-rays that showed hip or knee arthritis or OA but did not report a doctor’s diagnosis for these conditions were excluded from the analyses (*n*=7, <1% for hip arthritis or hip OA; *n*=5, <1% for knee arthritis or knee OA).

### Instruments

Three validated instruments were included in the study questionnaire. The Western Ontario and McMaster Universities Osteoarthritis (WOMAC) Index is a disease-specific measure of health status widely used in OA research [34], with demonstrated validity, reliability and responsiveness [35,36]. It produces pain, stiffness and physical function subscale scores commonly transformed to 0 (best health) to 100 (worst health) scales. Among participants with hip or knee arthritis or OA, the total WOMAC score was used to classify joint disease severity, as reported previously [37]. A WOMAC score <7 was considered to be asymptomatic joint disease, 7–38 was considered to be mild-moderate disease and ≥39 was considered to be severe joint disease. The Assessment of Quality of Life (AQoL) instrument is an Australian generic measure of HRQoL which was developed using the World Health Organization’s (WHO) definition of health and classification of impairments, disabilities and handicaps [38,39]. The four dimensions covered by this instrument include Independent Living, Social Relationships, Physical Senses and Psychological Wellbeing. The AQoL instrument has demonstrated good internal consistency, and face, content and construct validity [40]. It has been used in arthritis research [40,41] and Australian normative data are available [39]. The AQoL instrument produces a utility score ranging from −0.04 (worst HRQoL) to 1.00 (full HRQoL). The Hip and Knee Multi-Attribute Prioritisation Tool (MAPT) is a measure of OA disease severity and need for joint replacement surgery. The MAPT is used in all public hospitals in the state of Victoria to specifically facilitate prioritisation and care pathway triage for people with OA who may need hip or knee joint replacement. Although it has been shown to correlate with the WOMAC Index [42], the MAPT captures additional information relevant to hip and knee OA. Developed through intensive stakeholder consultation, it covers a range of constructs important to both patients and orthopaedic surgeons including pain, self-care activities, mental health, overall worsening and the ability to manage financially, care for others and maintain relationships. The MAPT has demonstrated construct validity and test-retest reliability in a large validation study [42] and produces a score ranging from 0 (least disease severity) to 100 (greatest severity).

Other self-reported data included country of birth, marital status, highest level of education, height, weight, paid and unpaid employment and premature exit from the workforce. BMI was calculated using self-reported height and weight and classified into underweight/normal weight (BMI ≤24.99 kg/m^2^), overweight (BMI 25–29.99) and obese categories (BMI ≥30), according to World Health Organization definitions [43]. Residential location was classified as metropolitan or provincial/rural based on AEC ratings for each federal electoral division [44]. Socioeconomic status was approximated using postcodes to link to the Australian Socio-Economic Indexes for Areas (SEIFA) 2006 Index of Relative Socio-Economic Advantage and Disadvantage [45]. The first SEIFA decile represents geographical areas with the greatest socioeconomic disadvantage while the tenth decile represents areas with the greatest advantage.

### Statistical analysis

Analyses were undertaken using SPSS 18.0 and Stata 10.1. Only limited demographic information (sex, age group, socioeconomic status and location) was available for non-participants; chi-square tests were used to evaluate differences between participants and non-participants.

Based on affirmative responses to the screening questions, the overall prevalence of hip or knee arthritis or OA and Clopper-Pearson confidence interval estimates were calculated. Prevalence was also calculated according to sex, age, BMI, education, marital status, country of birth, location and socioeconomic status (Additional file 1). Multiple binary logistic regression was used to generate adjusted odds ratios for the odds of hip and knee arthritis and OA, with demographic and socioeconomic variables entered simultaneously as predictors. Reference categories included male sex, age <50 years, BMI ≤24.99 kg/m^2^ (underweight/normal weight), primary school or less level of education, married/living with partner, born in Australia, residing in a metropolitan location and first SEIFA decile (greatest socioeconomic disadvantage) (Additional file 1).

For participants who reported hip or knee arthritis or OA, analysis of covariance (ANCOVA) was used to evaluate associations between BMI category and pain, stiffness, physical function and HRQoL (with adjustment for age and sex). Post-hoc tests used a Bonferroni adjustment for multiple comparisons. Kruskal-Wallis tests were used to evaluate the relationship between BMI category and disease severity as the distribution of MAPT scores was severely skewed and this was not improved by log transformation.

## Results

### Participants

Questionnaires were returned by 1,158 individuals (23% response rate). Limited information was available for non-participants: questionnaires were returned for 91 individuals (2%) due to incorrect address details, 45 individuals (1%) declined to participate and 1 had died. One respondent was found to be aged less than 39 years (having been incorrectly listed in the 69–73 year age category in the federal electoral roll extract) and was subsequently excluded from the analyses. Age data for all analyses were calculated based on self-reported date of birth from the study questionnaire.

Response rates by state ranged from 15% to 31%. The Northern Territory had the lowest response rate, as well as the highest proportion of return to sender mail; this may relate to the remoteness of many areas in the territory and the larger indigenous population.

### Comparison of participants and non-participants

Although absolute differences between participants and non-participants were small, a greater proportion of participants were female (57% vs 52% for non-participants, chi-square=8.3, p<0.01) and living in areas with the highest socioeconomic advantage (17% vs 14% for non-participants, chi-square=22.9, p=0.01). A lower proportion of younger age groups was also seen among participants (14% aged 39–43 years and 15% aged 44–48 years vs 21% each for non-participants, chi-square=90.5, p<0.01). Sixty-seven per cent in both groups lived in metropolitan areas (chi-square=0.0, p=0.90).

### Demographics

The demographic characteristics of the sample are summarized in Table 1.

**Table 1:** Demographic characteristics of study participants

**Characteristic**	Overall sample (*n*=1157)*
Age (years), median (IQR)	57 (48–69)
Female, *n* (%)	656 (57)
Body Mass index (BMI) (kg/m^2^), *n* (%)	
Underweight / normal weight (BMI ≤24.99 kg/m^2^)	430 (37)
Overweight (BMI 25–29.99 kg/m^2^)	383 (33)
Obese (BMI ≥30 kg/m^2^)	248 (21)
Married or living with partner, *n* (%)	924 (80)
Living in metropolitan area, *n* (%)	775 (67)
SEIFA^†^ decile, *n* (%)	
First (greatest socioeconomic disadvantage)	56 (5)
Tenth (greatest socioeconomic advantage)	200 (17)
Highest level of education completed, *n* (%)	
Primary school or less	54 (5)
Year 7-10	263 (23)
Year 11-12	213 (18)
Trade / technical education	260 (23)
University	356 (31)
Australian-born, *n* (%)	894 (77)
English as main language spoken at home, *n* (%)	1104 (95)
Paid work, *n* (%)	
Paid employment	651 (56)
Retired	426 (37)
Unemployed	58 (5)
Stopped work due to hip or knee arthritis / OA	7 (<1)
Unpaid work, *n* (%)	
Currently does unpaid work	335 (29)
Unable to do unpaid work due to hip or knee arthritis / OA	33 (3)
Does not do unpaid work for other reasons	713 (62)

*Total numbers for characteristics may not equal 1157 due to missing responses.

^†^Australian Socio-Economic Indexes for Areas 2006 Index of Relative Socio-Economic Advantage and Disadvantage.

### Overall prevalence of hip and knee arthritis and osteoarthritis

Eighty-three participants reported having ever been told by a doctor or other health professional that they had hip arthritis (7%, 95%CI 6%-9%), and 57 reported hip OA (5%, 95%CI 4%-6%). There was some overlap between the hip arthritis and hip OA groups; of those who had been told they had hip arthritis, 57% (*n*=47) also reported hip OA. Of those with hip arthritis or hip OA (*n*=93), 8% were classified as asymptomatic, 45% as mild to moderate and 40% as severe; WOMAC scores were missing for the remaining 8%.

One hundred and sixty-nine participants reported having been told they had knee arthritis (15%, 95%CI 13%-17%) and 98 reported knee OA (8%, 95%CI 7%-10%). Of those who reported knee arthritis, 41% (*n*=69) also said they had been diagnosed with knee OA. Of those with knee arthritis or knee OA (*n*=198), 15% were classified as asymptomatic, 52% as mild to moderate and 27% as severe; WOMAC scores were missing for the remaining 7%.

### Overweight and obesity and odds of hip arthritis and osteoarthritis

After adjusting for demographic characteristics, obesity was associated with a higher prevalence of hip arthritis (Table 2). Participants who were obese were more than twice as likely to have hip arthritis (adjusted OR (AOR)=2.18, 95%CI 1.17-4.06), compared with those classified as underweight/normal weight. A higher prevalence of hip arthritis was also associated with increasing age (Table 2). Compared with the youngest age group, participants aged 80 years and over had the greatest odds of having hip arthritis (AOR=6.86, 95%CI 2.54-18.50), followed by those aged 70–79 years (AOR=5.13, 95%CI 2.29-11.49).

**Table 2:** Prevalence and odds of hip arthritis and osteoarthritis according to demographic characteristics

	**Hip arthritis**			**Hip osteoarthritis**		
**Characteristic**	**Prevalence*n*(%)**	**Unadjusted OR (95%CI)**	Adjusted OR* (95%CI)	**Prevalence*n*(%)**	**Unadjusted OR (95%CI)**	Adjusted OR* (95%CI)
Sex						
Male (*n=*500)	19 (4)	1.00	1.00	13 (3)	1.00	1.00
Female (*n=*656)	64 (10)	2.74 (1.62-4.63)	3.70 (1.98-6.91)	44 (7)	2.69 (1.43-5.05)	3.39 (1.59-7.22)
Age group						
<50 years (*n=*363)	14 (4)	1.00	1.00	11 (3)	1.00	1.00
50-59 years (*n=*290)	10 (3)	0.89 (0.39-2.04)	0.97 (0.39-2.41)	3 (1)	0.33 (0.09-1.21)	0.37 (0.10-1.39)
60-69 years (*n=*230)	17 (7)	1.99 (0.96-4.12)	2.01 (0.87-4.61)	15 (7)	2.23 (1.01-4.95)	1.95 (0.78-4.88)
70-79 years (*n=*178)	29 (16)	4.89 (2.51-9.51)	5.13 (2.29-11.49)	23 (13)	4.81 (2.29-10.11)	4.35 (1.79-10.56)
≥80 years (*n=*82)	13 (16)	4.77 (2.15-10.59)	6.86 (2.54-18.50)	5 (6)	2.13 (0.72-6.32)	1.89 (0.50-7.18)
Body Mass Index (BMI)						
Underweight / normal weight (*n=*430)	28 (7)	1.00	1.00	18 (4)	1.00	1.00
Overweight (*n=*383)	20 (5)	0.79 (0.44-1.43)	1.00 (0.53-1.89)	16 (4)	1.00 (0.50-1.98)	1.17 (0.56-2.44)
Obese (*n=*248)	26 (10)	1.68 (0.96-2.93)	2.18 (1.17-4.06)	16 (6)	1.58 (0.79-3.16)	1.62 (0.77-3.45)
Highest level of education						
Primary school or less (*n=*54)	8 (15)	1.00	1.00	6 (11)	1.00	1.00
Year 7–10 (*n=*263)	31 (12)	0.75 (0.32-1.74)	1.01 (0.37-2.76)	19 (7)	0.62 (0.23-1.62)	0.62 (0.20-1.94)
Year 11–12 (*n=*213)	12 (6)	0.34 (0.13-0.87)	0.53 (0.17-1.64)	10 (5)	0.39 (0.13-1.12)	0.49 (0.14-1.73)
Trade / technical (*n=*260)	15 (6)	0.34 (0.14-0.86)	0.74 (0.25-2.18)	10 (4)	0.31 (0.11-0.90)	0.64 (0.19-2.17)
University (*n=*356)	17 (5)	0.28 (0.12-0.69)	0.67 (0.22-1.99)	12 (3)	0.27 (0.10-0.77)	0.47 (0.14-1.62)
Marital status						
Married / living with partner (*n=*924)	61 (7)	1.00	1.00	41 (4)	1.00	1.00
Single / divorced / widowed (*n=*222)	22 (10)	1.56 (0.94-2.61)	0.94 (0.50-1.76)	16 (7)	1.70 (0.93-3.08)	1.17 (0.57-2.41)
Country of birth						
Australia (*n=*894)	59 (7)	1.00	1.00	42 (5)	1.00	1.00
Other (*n=*255)	24 (9)	1.47 (0.89-2.41)	1.73 (0.98-3.07)	15 (6)	1.27 (0.69-2.33)	1.25 (0.62-2.49)
Location						
Metropolitan (*n*=775)	56 (7)	1.00	1.00	40 (5)	1.00	1.00
Provincial or rural (*n*=381)	27 (7)	0.98 (0.61-1.57)	0.70 (0.36-1.35)	17 (4)	0.86 (0.48-1.53)	0.79 (0.37-1.71)

OR: odds ratio; 95%CI: 95% confidence interval.

*Adjusted values were derived from multiple binary logistic regression models with sex, age group, BMI, highest level of education, marital status, country of birth, location and SEIFA decile entered simultaneously as predictors.

Socioeconomic status (SEIFA) data for hip arthritis and osteoarthritis are presented in the Additional file 1 (Table A1).

For hip OA, no association between obesity and prevalence was observed (Table 2). An increased prevalence of hip OA was associated with being female (AOR=3.39, 95%CI 1.59-7.22) and aged 70–79 years (AOR=4.35, 95%CI 1.79-10.56).

### Overweight and obesity and odds of knee arthritis and osteoarthritis

Higher BMI was strongly associated with an increased prevalence of knee arthritis (Table 3). After adjusting for other demographic characteristics, participants who were overweight were more likely to report knee arthritis (AOR 1.87, 95%CI 1.14-3.07) while those who were obese had the highest odds (AOR=5.47, 95%CI 3.35-8.95). The prevalence of knee arthritis also increased substantially with age; those aged 70–79 years and 80 years or over had the greatest odds (AOR=3.16, 95%CI 1.67-5.99 and AOR=5.26, 95%CI 2.44-11.35, respectively). A lower prevalence of knee arthritis was seen among participants who had completed high school (AOR=0.25, 95%CI 0.11-0.60), trade or technical qualifications (AOR=0.28, 95%CI 0.13-0.65) or university (AOR=0.34, 95%CI 0.15-0.78), compared to those who had completed primary school or less.

**Table 3:** Prevalence and odds of knee arthritis and osteoarthritis according to demographic characteristics

	**Knee arthritis**			**Knee osteoarthritis**		
**Characteristic**	**Prevalence*n*(%)**	**Unadjusted OR (95%CI)**	Adjusted OR* (95%CI)	**Prevalence*n*(%)**	**Unadjusted OR (95%CI)**	Adjusted OR* (95%CI)
Sex						
Male (*n=*500)	71 (14)	1.00	1.00	36 (7)	1.00	1.00
Female (*n=*656)	98 (15)	1.07 (0.77-1.49)	1.15 (0.77-1.71)	62 (9)	1.34 (0.87-2.06)	1.51 (0.91-2.52)
Age group						
<50 years (*n=*363)	25 (7)	1.00	1.00	15 (4)	1.00	1.00
50-59 years (*n=*290)	39 (13)	2.10 (1.24-3.55)	1.95 (1.09-3.48)	18 (6)	1.55 (0.77-3.13)	1.36 (0.64-2.88)
60-69 years (*n=*230)	37 (16)	2.60 (1.52-4.45)	1.99 (1.08-3.66)	31 (13)	3.65 (1.92-6.93)	2.76 (1.34-5.69)
70-79 years (*n=*178)	45 (25)	4.67 (2.75-7.92)	3.16 (1.67-5.99)	26 (15)	4.00 (2.06-7.76)	2.78 (1.26-6.11)
≥80 years (*n=*82)	22 (27)	5.03 (2.66-9.50)	5.26 (2.44-11.35)	7 (9)	2.29 (0.90-5.81)	1.99 (0.65-6.08)
Body Mass Index (BMI)						
Underweight / normal weight (*n=*430)	36 (8)	1.00	1.00	15 (3)	1.00	1.00
Overweight (*n=*383)	49 (13)	1.61 (1.02-2.54)	1.87 (1.14-3.07)	27 (7)	2.10 (1.10-4.00)	2.11 (1.07-4.15)
Obese (*n=*248)	68 (27)	4.21 (2.71-6.54)	5.47 (3.35-8.95)	49 (20)	6.83 (3.74-12.48)	7.35 (3.85-14.02)
Highest level of education						
Primary school or less (*n=*54)	19 (35)	1.00	1.00	10 (19)	1.00	1.00
Year 7–10 (*n=*263)	58 (22)	0.50 (0.27-0.95)	0.56 (0.26-1.21)	30 (11)	0.55 (0.25-1.21)	0.50 (0.19-1.32)
Year 11–12 (*n=*213)	24 (11)	0.22 (0.11-0.45)	0.25 (0.11-0.60)	13 (6)	0.28 (0.11-0.67)	0.34 (0.12-0.98)
Trade / technical (*n=*260)	27 (10)	0.20 (0.10-0.40)	0.28 (0.13-0.65)	16 (6)	0.28 (0.12-0.65)	0.36 (0.13-1.01)
University (*n=*356)	40 (11)	0.22 (0.11-0.42)	0.34 (0.15-0.78)	28 (8)	0.36 (0.16-0.79)	0.40 (0.15-1.11)
Marital status						
Married / living with partner (*n=*924)	119 (13)	1.00	1.00	67 (7)	1.00	1.00
Single / divorced / widowed (*n=*222)	47 (21)	1.83 (1.26-2.66)	1.50 (0.96-2.36)	29 (13)	1.98 (1.24-3.14)	2.11 (1.23-3.62)
Country of birth						
Australia (*n=*894)	131 (15)	1.00	1.00	70 (8)	1.00	1.00
Other (*n=*255)	37 (15)	0.98 (0.66-1.46)	1.16 (0.74-1.82)	27 (11)	1.39 (0.87-2.23)	1.62 (0.95-2.78)
Location						
Metropolitan (*n*=775)	113 (15)	1.00	1.00	67 (9)	1.00	1.00
Provincial or rural (*n*=381)	56 (15)	1.01 (0.72-1.44)	0.88 (0.54-1.44)	31 (8)	0.94 (0.60-1.46)	1.00 (0.54-1.85)

OR: odds ratio; 95%CI: 95% confidence interval.

*Adjusted values were derived from multiple binary logistic regression models with sex, age group, BMI, highest level of education, marital status, country of birth, location and SEIFA decile entered simultaneously as predictors.

Socioeconomic status (SEIFA) data for knee arthritis and osteoarthritis are presented in the Additional file 1 (Table A2).

Similar to the knee arthritis group, overweight and obesity were clearly associated with an increased prevalence of knee OA (Table 3). Participants who were overweight had increased odds of having knee OA (AOR=2.11, 95%CI 1.07-4.15) while the obese group had the highest odds (AOR=7.35, 95%CI 3.85-14.02). The prevalence of knee OA was also higher in those aged 60–69 years (AOR=2.76, 95%CI 1.34-5.69) and 70–79 years (AOR=2.78, 95%CI 1.26-6.11). Participants who were single, divorced or widowed were more likely to have knee OA (AOR=2.11, 95%CI 1.23-3.62) while those who had completed high school were less likely to have knee OA (AOR 0.34, 95%CI 0.12-0.98).

### Impact of overweight and obesity in hip arthritis and osteoarthritis

Table 4 shows that among participants with hip arthritis, pain increased significantly with greater BMI (F=5.35, p=0.01). Post-hoc tests showed that participants who were obese had the highest pain (adjusted mean 44.3, 95%CI 34.3-54.4), compared to those classified as underweight/normal weight (adjusted mean 23.1, 95%CI 13.8-32.4). A similar pattern was seen for stiffness; those who were obese reported the greatest stiffness, compared to individuals in the underweight/normal weight group (F=3.66, p=0.03). Post-hoc analyses indicated that participants in both the overweight and obese groups had worse physical function (adjusted mean 43.8, 95%CI 32.1-55.5 and 45.5, 95%CI 35.2-55.7, respectively), compared to those in the underweight/normal weight group (adjusted mean 23.8, 95%CI 14.1-33.4).

**Table 4:** Impact of overweight and obesity

**Outcome**	***n***	**Underweight or normal weight**	***n***	**Overweight**	***n***	**Obese**	**p**
Hip arthritis							
WOMAC pain	28	23.1 (13.8-32.4)	18	40.8 (29.4-52.2)	25	44.3 (34.3-54.4)	0.01
WOMAC stiffness	28	31.7 (22.3-41.1)	18	43.9 (32.3-55.5)	26	50.4 (40.4-60.3)	0.03
WOMAC function	27	23.8 (14.1-33.4)	18	43.8 (32.1-55.5)	25	45.5 (35.2-55.7)	0.01
AQoL	28	0.66 (0.55-0.77)	18	0.48 (0.34-0.62)	25	0.43 (0.31-0.55)	0.02
MAPT	26	0.0 (0.0-7.5)	18	8.1 (0.0-38.6)	25	14.1 (2.3-55.7)	0.01
Hip osteoarthritis							
WOMAC pain	18	24.2 (11.9-36.5)	14	34.0 (19.8-48.2)	15	50.4 (36.6-64.2)	0.03
WOMAC stiffness	18	34.7 (23.4-46.1)	14	41.6 (28.5-54.6)	16	53.5 (41.2-65.8)	0.09
WOMAC function	18	27.7 (15.4-40.0)	14	39.2 (25.0-53.4)	15	52.7 (38.9-66.6)	0.03
AQoL	18	0.60 (0.47-0.73)	14	0.56 (0.41-0.71)	15	0.32 (0.18-0.47)	0.02
MAPT	16	2.2 (0.0-17.4)	14	3.8 (1.9-27.8)	15	39.7 (9.6-58.6)	0.01
Knee arthritis							
WOMAC pain	34	24.2 (16.1-32.3)	48	25.9 (19.1-32.6)	65	34.0 (28.2-39.8)	0.09
WOMAC stiffness	34	25.6 (17.2-34.0)	49	30.1 (23.1-37.0)	66	40.6 (34.6-46.7)	0.01
WOMAC function	33	20.7 (12.7-28.7)	48	26.6 (20.0-33.2)	65	35.9 (30.2-41.6)	0.01
AQoL	35	0.68 (0.59-0.77)	49	0.64 (0.57-0.72)	65	0.59 (0.52-0.65)	0.24
MAPT	34	0.0 (0.0-5.3)	44	0.0 (0.0-9.4)	58	6.1 (0.0-23.7)	0.01
Knee osteoarthritis							
WOMAC pain	15	23.4 (10.9-35.8)	26	24.0 (14.6-33.4)	47	40.1 (33.0-47.1)	0.01
WOMAC stiffness	15	24.0 (11.3-36.6)	27	29.2 (19.8-38.5)	48	44.3 (37.3-51.4)	0.01
WOMAC function	14	20.1 (7.6-32.6)	27	24.0 (15.1-33.0)	47	40.6 (33.7-47.4)	<0.01
AQoL	15	0.65 (0.52-0.79)	27	0.63 (0.53-0.73)	46	0.53 (0.46-0.61)	0.20
MAPT	15	2.5 (0.0-6.6)	26	2.5 (0.0-7.2)	43	12.9 (0.0-39.9)	0.01

WOMAC and AQoL data presented as adjusted mean (95%CI); p-values from ANCOVA with adjustment for age and sex.

MAPT data presented as median (interquartile range); p-values from Kruskal-Wallis test.

Higher WOMAC score indicates higher pain, greater stiffness or worse function; higher MAPT score indicates greater disease severity; lower AQoL score indicates lower HRQoL.

Regardless of BMI category, the average HRQoL of participants with hip arthritis was well below Australian population norms (mean AQoL 0.83, 95%CI 0.82-0.84, minimal important difference ~0.06 AQoL units [39]). Further deterioration in HRQoL was evident with increasing BMI (F=4.44, p=0.02); there was a significant difference in HRQoL between participants who were obese (adjusted mean 0.43, 95%CI 0.31-0.55) and those classified as underweight/normal weight (adjusted mean 0.66, 95%CI 0.55-0.77). Increasing BMI was also associated with greater disease severity (Kruskal-Wallis chi-square=9.9, p=0.01). Participants who were obese had the greatest severity (median 14.1, IQR 2.3-55.7), followed by those who were overweight (median 8.1, IQR 0.0-38.6). Those classified as underweight/normal weight had the least severe disease (median 0.0, IQR 0.0-7.5).

For participants with hip OA, increased pain was also associated with greater BMI (F=4.04, p=0.03). Those classified as obese had higher pain (adjusted mean 50.4, 95%CI 36.6-64.2), compared to participants in the underweight/normal weight group (adjusted mean 24.2, 95%CI 11.9-36.5). There was a trend towards greater stiffness with increasing BMI but this was not significant (F=2.55, p=0.09). Obese participants also had worse physical function (F=3.68, p=0.03), markedly lower HRQoL (F=4.48, p=0.02) and greater disease severity (Kruskal-Wallis chi square=9.5, p=0.01), compared to those classified as underweight/normal weight (Table 4). Those with hip OA who were obese had extremely low HRQoL, compared to population norms (mean AQoL score 0.32, 95%CI 0.18-0.47).

### Impact of overweight and obesity in knee arthritis and osteoarthritis

In knee arthritis, greater stiffness was associated with increased BMI (F=4.76, p=0.01); participants who were obese reported more stiffness (adjusted mean 40.6, 95%CI 34.6-46.7) than those in the underweight/normal weight group (adjusted mean 25.6, 95%CI 17.2-34.0). Participants who were obese also had worse function (adjusted mean 35.9, 95%CI 30.2-41.6), compared to those classified as underweight/normal weight (adjusted mean 20.7, 95%CI 12.7-28.7). Participants in the obese group had, on average, greater disease severity (median 6.1, IQR 0.0-23.7; Kruskal-Wallis chi-square=8.6, p=0.01).

Among participants with knee OA, those classified as obese had higher pain (adjusted mean 40.1, 95%CI 33.0-47.1) than participants who were overweight (adjusted mean 24.0, 95%CI 14.6-33.4). Participants who were obese also had greater stiffness (F=5.48, p=0.01) and worse physical function (F=6.39, p<0.01), compared to both the overweight and underweight/normal weight groups. Obesity was also associated with increased disease severity (Kruskal-Wallis chi-square=10.2, p=0.01).

## Discussion

Using a national, population health approach, this research is the first to explore the relationship between obesity and the burden of hip and knee joint disease in Australia. This study has shown that overweight was associated with greater likelihood of having knee arthritis and knee OA, and that obesity was associated with the highest odds of hip arthritis, knee arthritis and knee OA. Regardless of diagnosis, obesity was consistently associated with reduced physical function and greater disease severity, and people with hip arthritis or hip OA who were obese also experienced very low HRQoL, compared with population norms. Generating data on both odds of joint disease *and* the personal impact of concurrent obesity and joint disease (in terms of pain, function, HRQoL and disease severity), this study provides new information which is relevant for clinicians, health planners and policy makers. Our use of a population-based sampling frame is a key strength which should improve generalisability of the findings across the disease severity spectrum, in comparison to clinically-based studies which typically involve people with more severe joint disease who are already receiving care. As in many developed countries, obesity poses a significant public health problem for Australia, with recent data indicating that 68% and 55% of men and women, respectively, are overweight or obese [46]. Arthritis (including OA) and obesity have both been designated as National Health Priority Areas in Australia [47], reflecting their current and anticipated burden to the community. Reducing the burden of concurrent obesity and joint disease will undoubtedly require a multi-faceted approach which incorporates primary and secondary prevention programs, improved public education about weight loss, physical activity and joint protection, and appropriate support for clinicians to provide effective education and evidence-based care.

While comparisons across prevalence studies are difficult due to variation in case definitions (e.g. ‘symptomatic’, ‘radiographic’ and ‘doctor-diagnosed’ arthritis or OA), our findings are in line with studies from the US, the Netherlands, England and Norway which have consistently demonstrated that obesity is associated with an increased risk of knee OA [7,10,12,48,49]. The association between obesity and prevalence of hip OA is less clear [6]; some studies have identified a greater risk with increased BMI (AOR 1.72, 95%CI 1.08-2.74 [49]) while others found no link (AOR 1.0, 95%CI 0.7-1.5 [10] and 1.11, 95%CI 0.41-2.97 [11]). We did not find a significant association between obesity and odds of hip OA (AOR 1.62, 95%CI 0.77-3.45), but these analyses were based on a relatively small sample with limited power. We observed that obesity was associated with increased odds of hip arthritis, although this was considerably lower than for knee arthritis or knee OA (AOR 2.18 vs 5.47 and 7.35, respectively). It has been suggested that excessive weight may exert a greater impact on the knee joint through biomechanical factors, to which the more stable hip joint might be less susceptible [50].

Although the specific pattern of impairment varied somewhat according to diagnosis, this study provides new evidence of the association between obesity and burden of arthritis and OA. Among those with arthritis or OA, obese individuals reported more pain (for hip arthritis, hip OA and knee OA), greater stiffness (for hip arthritis, knee arthritis and knee OA), worse function (for all diagnoses), lower HRQoL (for hip arthritis and hip OA) and greater disease severity (for all diagnoses). To date, population-based research into the personal impact of obesity and hip or knee OA has been limited. Most studies have involved patients in clinical settings [17,21-24,51], including those with end-stage OA awaiting hip replacement [52]. However, the observed relationships between poorer function and obesity are consistent with previous studies that have reported reduced physical function [22,52,53], lower physical activity [17] and greater disability [24,27,28] with increasing BMI. Further research is needed to investigate specific mechanisms for reduced function and identify potential avenues for rehabilitation. An important finding was that although HRQoL was lower than Australian norms across each diagnosis group [39], obese individuals with hip arthritis or hip OA clearly had very poor HRQoL. This highlights the substantial personal impact of concurrent obesity and hip joint disease. Lower quality of life was also evident in a large study of primary care patients with hip or knee OA who were overweight or obese [17], but separate hip and knee analyses were not reported. We did not find an association between obesity and HRQoL in knee arthritis or knee OA, similar to a population-based study of Japanese women [54]. Some clinical studies have looked at associations between obesity and indicators of disease severity (e.g. WOMAC) [22,23], and our use of the MAPT instrument provides further insight into this relationship. In view of rising obesity rates, the observed relationship between obesity and disease severity may have potential implications for health services demand. However, our analyses indicate substantial within-group variability and further investigation should involve larger samples.

While an individual’s understanding of their arthritis type may be limited, our methods were similar to other population-based studies (including the Australian National Health Survey) which have asked participants specifically about ‘osteoarthritis’ [11,55]. As OA is the most common form of arthritis [56], our use of the terms ‘arthritis’ *and* ‘osteoarthritis’ in the screening questions should have captured the majority of hip and knee OA cases. However, we acknowledge that self-reported data can be inaccurate. Our analyses may also underestimate the prevalence of doctor-diagnosed arthritis and OA, given the additional participants who reported positive x-rays but did not report having been told they had arthritis or OA. We excluded this group from our analyses, in line with previous recommendations [57].

A major strength of our study was the use of federal electoral rolls to sample the Australian adult population; however, the main limitation is the low response rate (23%). Australian government-funded population health surveys commonly use Computer Assisted Telephone Interviews or face-to-face interviews and have reported participation rates of approximately 65% [13,14]. It is possible that people are more likely to participate in government-branded research [58] and there may be a greater community awareness of these ongoing surveys. Comparing response rates between population-based studies of joint disease is difficult, due to differences in recruitment scope (e.g. local, regional or national sampling), differences in the way potential participants are approached (e.g. with a preliminary telephone call or introductory letter) and possible cultural differences between settings which could affect research participation. We found that younger individuals were less likely to participate, similar to a population-based study of knee, hip and hand OA in Norway which also found that younger individuals were less likely to respond to mailed questionnaires [49]. Although differential response rates according to age could potentially introduce bias, we do not expect this to have impacted significantly on our results given that our data show the prevalence of joint disease to be low among younger people. Additionally, we included age as a predictor in our regression models and as a covariate in the ANCOVA analyses. Despite the response rate, the sample can be considered broadly representative of the Australian population across several key characteristics. BMI distribution was similar to that reported by the Australian Bureau of Statistics (39% underweight/normal weight, 37% overweight and 24% obese [46]. Sixty-seven per cent of the sample lived in a metropolitan area, compared with 58% from overall Australian electoral roll data [59]. Although the study sample comprised a greater proportion of people from higher SES areas (21% and 17% living in the ninth and tenth SEIFA deciles, respectively), higher education status closely reflected Australian government data on educational attainment [60]. In a 2011 report, 62% of Australians aged 35–64 years had a non-school qualification (eg technical education or university), which is comparable to that reported by our sample (61% when limited to those aged less than 65 years). Additionally, average HRQoL for the overall sample was similar to Australian population norms (mean (SD) AQoL score for sample 0.81 (0.21) versus population norm 0.83 (0.20) [39]).

We also acknowledge that self-reported height and weight information may underestimate true BMI [15,61] and that our cross-sectional design precludes causal inferences. It is therefore unknown, for example, whether obesity led to reduced physical function or if lower function due to OA resulted in weight gain. Finally, although we included some covariates (age and gender) in the HRQoL analyses, it is possible that the relationship between BMI and HRQoL may be affected by other factors, such as mood or depression, although we did not collect this information. This study has a number of strengths including the use of population-based sampling to include people with a range of joint disease severity, recruitment across all 8 Australian states and territories to maximise generalisability, and the evaluation of both prevalence *and* personal impact (across a range of constructs relevant to people with joint disease including pain, function, HRQoL and disease severity). It has generated the first national data on the obesity-related burden of hip and knee joint disease in Australia, and provides new evidence from outside the US on the relationships between obesity and key indicators of wellbeing relevant to people with hip and knee joint disease.

## Conclusions

This national study has shown that obesity was associated with an increased burden of hip and knee joint disease, as evidenced by higher prevalence and greater impairment in key indicators of wellbeing. In particular, people with hip arthritis or hip OA who were obese had extremely low HRQoL. While relationships between obesity, pain, function and HRQoL are likely to be complex, these data highlight the need for public health interventions that consider overweight and obesity as both primary and secondary intervention targets for people with hip or knee joint disease.

## Abbreviations

ANCOVA: Analysis of covariance; AOR: Adjusted odds ratio; AQoL: Assessment of Quality of Life instrument; BMI: Body Mass Index; HRQoL: Health-Related Quality of Life; MAPT: Multi-Attribute Prioritisation Tool; OA: Osteoarthritis; OR: Odds ratio; SEIFA: >Australian Socio-Economic Indexes for Areas; WOMAC: Western Ontario and McMaster Universities Osteoarthritis Index.

## Competing interests

The authors declare that they have no competing interests.

## Authors’ contributions

Both authors contributed to the design of the study. Data collection and statistical analysis was undertaken by INA. Both authors contributed to drafting the manuscript and have approved the final manuscript.

## Pre-publication history

The pre-publication history for this paper can be accessed here:

http://www.biomedcentral.com/1471-2474/13/254/prepub

## Supplementary Material

Additional file 1 **This file presents data on the prevalence and odds of arthritis and osteoarthritis according to socioeconomic status, as referred to in the legends of Table **2** and Table **3**.** Click here for file
