# Supplementary material for: Obesity and increased burden of hip and knee joint disease in Australia: Results from a national survey
Source: BMC Musculoskelet Disord. 2012 Dec 20;13:254. doi: 10.1186/1471-2474-13-254 (PMC3564744; doi:10.1186/1471-2474-13-254)
Supplement: Additional file 1 — This file presents data on the prevalence and odds of arthritis and osteoarthritis according to socioeconomic status, as referred to in the legends of Table 2 and Table 3. [file 1471-2474-13-254-S1.doc]

**Additional file**

**Table A1. Prevalence and odds of hip arthritis and osteoarthritis according to socioeconomic status**

|  | **Hip arthritis** | | |  | **Hip osteoarthritis** | | |
| --- | --- | --- | --- | --- | --- | --- | --- |
| **Characteristic** | **Prevalence**  ***n* (%)** | **Unadjusted OR (95%CI)** | **Adjusted OR* (95%CI)** |  | **Prevalence**  ***n* (%)** | **Unadjusted OR (95%CI)** | **Adjusted OR* (95%CI)** |
| SEIFA^†^ decile |  |  |  |  |  |  |  |
| First (greatest disadvantage) (*n*=56) | 3 (5) | 1.00 | 1.00 |  | 3 (5) | 1.00 | 1.00 |
| Second (*n*=77) | 9 (12) | 2.34 (0.60-9.07) | 2.08 (0.46-9.34) |  | 4 (5) | 0.97 (0.21-4.51) | 1.02 (0.20-5.23) |
| Third (*n*=63) | 2 (3) | 0.58 (0.09-3.60) | 0.55 (0.08-3.70) |  | 2 (3) | 0.58 (0.09-3.60) | 0.66 (0.10-4.37) |
| Fourth (*n*=62) | 8 (13) | 2.62 (0.66-10.40) | 2.82 (0.63-12.55) |  | 8 (13) | 2.62 (0.66-10.40) | 3.09 (0.69-13.74) |
| Fifth (*n*=76) | 6 (8) | 1.51 (0.36-6.34) | 1.47 (0.32-6.71) |  | 5 (7) | 1.28 (0.29-5.60) | 1.28 (0.27-6.08) |
| Sixth (*n*=105) | 11 (10) | 2.07 (0.55-7.74) | 2.29 (0.55-9.48) |  | 3 (3) | 0.52 (0.10-2.66) | 0.41 (0.06-2.66) |
| Seventh (*n*=118) | 6 (5) | 0.95 (0.23-3.93) | 1.02 (0.22-4.69) |  | 4 (3) | 0.62 (0.13-2.87) | 0.70 (0.14-3.59) |
| Eighth (*n*=159) | 8 (5) | 0.94 (0.24-3.66) | 0.74 (0.16-3.56) |  | 5 (3) | 0.57 (0.13-2.48) | 0.48 (0.09-2.70) |
| Ninth (*n*=240) | 13 (5) | 1.02 (0.28-3.71) | 1.18 (0.29-4.88) |  | 10 (4) | 0.78 (0.21-2.91) | 0.90 (0.21-3.90) |
| Tenth (greatest advantage) (*n*=200) | 17 (9) | 1.64 (0.46-5.81) | 1.72 (0.41-7.20) |  | 13 (7) | 1.23 (0.34-4.47) | 1.18 (0.26-5.34) |

OR: odds ratio; 95%CI: 95% confidence interval

*Adjusted values were derived from multiple binary logistic regression models with sex, age group, BMI, highest level of education, marital status, country of birth, location and SEIFA decile entered simultaneously as predictors

^†^Australian Socio-Economic Indexes for Areas 2006 Index of Relative Socio-Economic Advantage and Disadvantage

**Table A2. Prevalence and odds of knee arthritis and osteoarthritis according to socioeconomic status**

|  | **Knee arthritis** | | |  | **Knee osteoarthritis** | | |
| --- | --- | --- | --- | --- | --- | --- | --- |
| **Characteristic** | **Prevalence**  ***n* (%)** | **Unadjusted OR (95%CI)** | **Adjusted OR* (95%CI)** |  | **Prevalence**  ***n* (%)** | **Unadjusted OR (95%CI)** | **Adjusted OR* (95%CI)** |
| SEIFA^†^ decile |  |  |  |  |  |  |  |
| First (greatest disadvantage) (*n*=56) | 9 (16) | 1.00 | 1.00 |  | 4 (7) | 1.00 | 1.00 |
| Second (*n*=77) | 15 (19) | 1.24 (0.50-3.07) | 1.04 (0.36-2.98) |  | 10 (13) | 1.94 (0.58-6.54) | 1.80 (0.46-7.09) |
| Third (*n*=63) | 9 (14) | 0.85 (0.31-2.33) | 0.67 (0.22-2.06) |  | 5 (8) | 1.14 (0.29-4.48) | 1.29 (0.30-5.56) |
| Fourth (*n*=62) | 13 (21) | 1.38 (0.54-3.55) | 1.16 (0.39-3.42) |  | 7 (11) | 1.75 (0.48-6.34) | 1.80 (0.44-7.44) |
| Fifth (*n*=76) | 12 (16) | 0.96 (0.37-2.46) | 0.67 (0.22-2.02) |  | 7 (9) | 1.34 (0.37-4.82) | 1.34 (0.32-5.55) |
| Sixth (*n*=105) | 13 (12) | 0.73 (0.29-1.83) | 1.02 (0.37-2.81) |  | 3 (3) | 0.38 (0.08-1.77) | 0.57 (0.12-2.86) |
| Seventh (*n*=118) | 13 (11) | 0.64 (0.26-1.60) | 0.51 (0.18-1.48) |  | 12 (10) | 1.47 (0.45-4.79) | 1.72 (0.47-6.26) |
| Eighth (*n*=159) | 17 (11) | 0.61 (0.26-1.47) | 0.86 (0.32-2.29) |  | 6 (4) | 0.51 (0.14-1.89) | 0.80 (0.20-3.24) |
| Ninth (*n*=240) | 39 (16) | 1.00 (0.45-2.21) | 1.30 (0.52-3.28) |  | 28 (12) | 1.74 (0.59-5.18) | 2.44 (0.71-8.39) |
| Tenth (greatest advantage) (*n*=200) | 29 (15) | 0.87 (0.38-1.96) | 1.20 (0.45-3.16) |  | 16 (8) | 1.13 (0.36-3.53) | 1.50 (0.40-5.66) |

OR: odds ratio; 95%CI: 95% confidence interval

*Adjusted values were derived from multiple binary logistic regression models with sex, age group, BMI, highest level of education, marital status, country of birth, location and SEIFA decile entered simultaneously as predictors

^†^Australian Socio-Economic Indexes for Areas 2006 Index of Relative Socio-Economic Advantage and Disadvantage
